# Thyroid Hormone and P-Glycoprotein in Tumor Cells

**DOI:** 10.1155/2015/168427

**Published:** 2015-03-19

**Authors:** Paul J. Davis, Sandra Incerpi, Hung-Yun Lin, Heng-Yuan Tang, Thangirala Sudha, Shaker A. Mousa

**Affiliations:** ^1^Department of Medicine, Albany Medical College, Albany, NY 12208, USA; ^2^Pharmaceutical Research Institute, Albany College of Pharmacy and Health Sciences, 1 Discovery Drive, Rensselaer, NY 12144, USA; ^3^Department of Sciences, University Roma Tre, 00146 Rome, Italy; ^4^PhD Program for Cancer Biology and Drug Discovery, College of Medical Science and Technology, Taipei Medical University, Taipei 110, Taiwan

## Abstract

P-glycoprotein (P-gp; multidrug resistance pump 1, MDR1; ABCB1) is a plasma membrane efflux pump that when activated in cancer cells exports chemotherapeutic agents. Transcription of the P-gp gene (*MDR1*) and activity of the P-gp protein are known to be affected by thyroid hormone. A cell surface receptor for thyroid hormone on integrin *α*v*β*3 also binds tetraiodothyroacetic acid (tetrac), a derivative of L-thyroxine (T_4_) that blocks nongenomic actions of T_4_ and of 3,5,3′-triiodo-L-thyronine (T_3_) at *α*v*β*3. Covalently bound to a nanoparticle, tetrac as nanotetrac acts at the integrin to increase intracellular residence time of chemotherapeutic agents such as doxorubicin and etoposide that are substrates of P-gp. This action chemosensitizes cancer cells. In this review, we examine possible molecular mechanisms for the inhibitory effect of nanotetrac on P-gp activity. Mechanisms for consideration include cancer cell acidification via action of tetrac/nanotetrac on the Na^+^/H^+^ exchanger (NHE1) and hormone analogue effects on calmodulin-dependent processes and on interactions of P-gp with epidermal growth factor (EGF) and osteopontin (OPN), apparently via *α*v*β*3. Intracellular acidification and decreased H^+^ efflux induced by tetrac/nanotetrac via NHE1 is the most attractive explanation for the actions on P-gp and consequent increase in cancer cell retention of chemotherapeutic agent-ligands of MDR1 protein.

## 1. Introduction

P-glycoprotein (P-gp; multidrug resistance protein 1, MDR1; ABCB1) is a plasma membrane efflux pump with broad ligand specificity in normal cells and in cancer cells [1]. A glycoprotein ATPase is responsible in cancer cells for the outward transport of a variety of chemotherapeutic agents and thus is a critical vehicle of chemoresistance. P-gp is subject to pharmacologic inhibition with a variety of agents, for example, the calcium channel blocker, verapamil [1, 2], and tyrosine kinase inhibitors [3]. The search for effective P-gp inhibitor drugs is active [4, 5]. Thyroid hormone, L-thyroxine (T_4_) or 3,3′,5-triiodo-L-thyronine (T_3_), is known to induce transcription of P-glycoprotein (*MDR1*) gene [6–8] and P-gp function [8]. We have shown that a thyroid hormone antagonist, tetraiodothyroacetic acid (tetrac), acting at the thyroid hormone-tetrac receptor on plasma membrane integrin *α*v*β*3, increases the intracellular residence time of doxorubicin in chemoresistant (doxorubicin-resistant) human breast cancer cells [9]. This is an index of inhibition of P-gp activity. Thus, in cancer cells, this function of the hormone supports drug resistance, whereas in nonmalignant cells, this action of the hormone may stimulate desirable efflux of toxic substances accumulated by the cells. In this review, we examine the mechanisms by which thyroid hormone, tetrac and nanoparticulate tetrac formulation (nanotetrac) that acts exclusively at integrin *α*v*β*3, may regulate P-gp function in cancer cells. The integrin is also known to regulate P-gp by other mechanisms [10].

## 2. Integrin *α*v*β*3 and Nongenomic Actions of Thyroid Hormone

Integrins are structural proteins of the plasma membrane that bind extracellular matrix (ECM) proteins and are integral to cell-cell adhesion and cell-ECM protein interactions. Among ECM protein ligands of various integrins are fibronectin, vitronectin, osteopontin (OPN), and von Willebrand factor [11]. Of more than 20 integrins, only *α*v*β*3 contains a receptor site for thyroid hormone [12]. *α*v*β*3 is amply expressed by tumor cells and rapidly dividing endothelial cells usually found supporting cancers. We have described cancer cell proliferation* in vitro* in response to T_4_ and T_3_ in a variety of human cells [13–15] and these hormones are proangiogenic by a variety of mechanisms [16–18]. Both actions are wholly dependent upon the hormone-tetrac receptor on integrin *α*v*β*3. Such actions of T_4_ and T_3_ at the integrin are termed nongenomic because they do not primarily require the interaction of nuclear thyroid hormone receptors (TRs) with T_3_, the definition of the genomic mechanism of hormone action [19]. T_4_ is active at the integrin and the affinity of the hormone receptor on *α*v*β*3 is higher for T_4_ than for T_3_; in contrast, T_4_ in genomic actions is a prohormonal source of T_3_ via deiodination.

Tetrac and nanotetrac inhibit binding of agonist thyroid hormone to the receptor on the ectodomain of *α*v*β*3. But, in the absence of T_4_ and T_3_, nanotetrac and tetrac have a set of novel proapoptotic and antiangiogenic actions [17]. These involve modulation of crosstalk between the integrin and adjacent vascular growth factor receptors, the promotion of apoptosis, and the disordering of transcription of genes important to cell survival pathways [17, 30]. Specifically, there is crosstalk between *α*v*β*3 and receptors for vascular endothelial growth factor (VEGF) [17] and epidermal growth factor (EGF) [17] that may be relevant to the *α*v*β*3-mediated effects of thyroid hormone on cellular retention of chemotherapeutic agents (see next section).

From the integrin, T_4_ can also alter intracellular trafficking and state of serine phosphorylation of TRs, of estrogen receptor-*α* (ER*α*), of signal transducing and activator of transcription (STAT) proteins, and of p53 [17]. These phosphorylation steps are dependent upon mitogen-activated protein kinase (MAPK; ERK1/2) and represent an interesting adjunctive interface of nongenomic actions with genomic actions of thyroid hormone. In human lung carcinoma cells that express ER*α*, T_4_ may be estrogen-like, supporting cell proliferation that is ER-dependent [15]. Migration of endothelial cells toward a vitronectin cue is also stimulated by T_4_ via *α*v*β*3 [18]. Fibroblast migration in an* in vitro* model of wound-healing is also stimulated by T_4_ at the cell surface hormone receptor (SA Mousa: unpublished observations). The state of the actin cytoskeleton is nongenomically regulated by T_4_ [31, 32], in part reflecting action of the hormone to increase the amount of fibrous (F) actin from the pool of available soluble actin.

Finally, thyroid hormone can nongenomically regulate the activities of several plasma membrane transport systems, including the sodium/proton (Na^+^/H^+^) exchanger (NHE1) or antiporter [33, 34], Na^+^, K^+^-ATPase [35, 36], and the glucose transport system [37]. The action on NHE1 contributes to regulation of intracellular pH (pHi). Inhibition of this integrin-mediated effect of thyroid hormone decreases cellular pHi and may permit modulation of activity of enzymes whose pH optima are physiologic or slightly alkaline. Increased activity of NHE1 will also decrease extracellular pH (pHe), an effect that may reduce cell uptake of certain chemotherapeutic agents [21]. The plasma membrane calcium pump (Ca^2+^-ATPase) is another ATPase whose transport activity is activated nongenomically by T_4_ [38–40].

## 3. Possible Mechanisms by Which Tetrac and Agonist Thyroid Hormone Cause Tumor Cell Retention of Chemotherapeutic Agents

When we studied doxorubicin-resistant human breast cancer (MCF-7/dox) cells* in vitro*, we confirmed shortened intracellular residence time of labeled doxorubicin in these cells [9]. Tetrac exposure significantly increased residence time of doxorubicin in MCF-7/dox cells. The residence time of etoposide and cisplatin in neuroblastoma and osteosarcoma cell lines was also increased by tetrac. Of importance here is that doxorubicin and etoposide are P-gp substrates, whereas cisplatin is not. P-gp may influence the activities of certain apoptosis-relevant proteins such as p53 and caspase-3 and thus increase cancer cell sensitivity to agents such as cisplatin that are not P-gp substrates [41]. This indicates that tetrac may inactivate mechanisms of resistance in addition to the efflux pump. In studies we have carried out [9], we found that tetrac did not alter cellular abundance of superoxide dismutase (SOD) or glutathione-S-transferase-*π* (GST-*π*) proteins that support chemoresistance in the MCF-7/dox cell line. The P-gp protein abundance was ample in resistant cells but undetectable in wild-type MCF-7 cells. We postulated that tetrac decreased the activity of the P-gp ATPase to cause increased residence time of doxorubicin and etoposide, because agonist thyroid hormones (T_4_ and T_3_) nongenomically increase the activity of a variety of plasma membrane pumps—including several ATPases—and tetrac blocks nongenomic actions of T_4_ and T_3_, which are agonists at their receptor on *α*v*β*3.

What are the molecular mechanisms that might be modulated by tetrac to result in decreased activity of P-gp and tumor cell retention of P-gp ligands such as doxorubicin and etoposide? Tetrac will block binding of thyroid hormone to integrin *α*v*β*3 and if transcription of* MDR1* is regulated from the cell surface, as is expression of a wide variety of genes [17, 30], then this action will decrease abundance of the protein in cancer cells. Thyroid hormone does increase transcription of* MDR1* [6–8, 42]. This effect of the hormone does not involve the pregnane X receptor/steroid and xenobiotic receptor (PXR/SXR) [42] that is usually implicated in* MDR1* gene expression, thus indicating the existence of one or more alternative pathways for regulation of* MDR1* expression. Gene expression modulation from the integrin by thyroid hormone and tetrac formulations may involve alteration of the states of phosphorylation and acetylation of certain intranuclear receptors, as well as regulation of coactivator/corepressor complex formation [17]. Thus, it is not surprising that the hormone can affect* MDR1* expression independently of PXR/SXR. Integrin *α*v*β*3 has recently been shown to affect* MDR1* expression by the phosphatidylinositol 3-kinase (PI3-K)/Akt pathway [10] that we have implicated in a variety of actions of thyroid hormone and tetrac initiated at this integrin [17].

**Figure 1:**
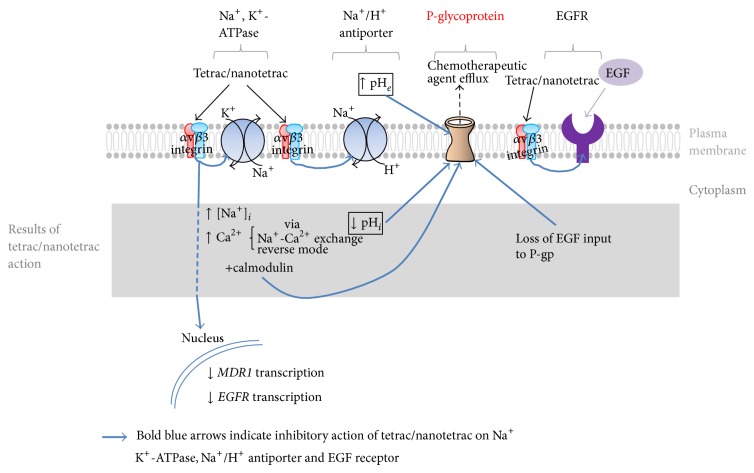
Schematic overview of possible mechanisms in tumor cells by which tetrac and nanotetrac may decrease function or abundance or both of P-gp. Postulated mechanisms are initiated at the thyroid hormone-tetrac receptor site on integrin *α*v*β*3 in the plasma membrane. An example shown is downregulation of the Na^+^/H^+^ antiporter by tetrac that results in decreased intracellular pH (pHi) and increased extracellular pH (pHe), both of which may serve to reduce P-gp function (see text). Another example is inhibition by tetrac of Na, K-ATPase, resulting in increased [Na^+^]_*i*_, reverse mode Na^+^/Ca^2+^ exchange, and increased [Ca^2+^]_*i*_. The latter, in conjunction with calmodulin, can downregulate P-gp activity. EGF is one of several extracellular factors that supports P-gp activity. Tetrac/nanotetrac may remove any contributions of EGF to P-gp activity by disrupting function of the plasma membrane EGF receptor (EGFR) or by decreasing* EGFR* gene expression. The figure also proposes that the decreased expression of the* MDR1* gene is initiated at integrin *α*v*β*3; this possibility has not yet been explored. The figure does not include factors such as osteopontin and VEGF that are also known to regulate P-gp and whose actions might be affected by tetrac/nanotetrac. These factors are discussed in the text.

Thyroid hormone also enhances function of the P-gp protein [8], but it is not yet known whether the latter effect is nongenomic in mechanism. Another possible mechanism of tetrac action on P-gp is sustained intracellular acidification, such as that induced pharmacologically with cariporide, an NHE1 inhibitor. This results in decreased P-gp activity [20] and also causes a reduction in* MDR1* (P-gp) gene expression and* MDR1* mRNA. Thyroid hormone acutely upregulates NHE1 activity and the inhibition of this nongenomic hormonal action by tetrac may result in a significant decrease in pHi [33, 34], away from the pH optimum of the pump. In addition, a consequence of the tetrac effect on NHE1 is failure of the antiporter to support the extracellular acidosis that favors P-gp transport function [43, 44]. It is important to point out that the bovine serum-supplemented medium that cancer cells require for growth contains ample amounts of T_4_ and T_3_. We can conclude that one mechanism by which tetrac may downregulate activity of P-gp in tumor cells is via its *α*v*β*3-dependent action on NHE1. Recent reviews of P-gp chemistry and conceptual approaches to the inhibition of efflux pump activity have not considered acidification of P-gp-containing cells [1, 4, 5] as a strategy. This omission presumably reflects an assumption that pharmacologic acidification will affect normal cells, as well as tumor cells. This need not be the case when the pharmacologic initiation site is a protein such as integrin *α*v*β*3 whose expression/activation is primarily by tumor cells and rapidly dividing endothelial cells. A summary of molecular mechanisms by which tetrac and nanotetrac may affect P-gp function or abundance is presented in Figure 1.

As noted above, thyroid hormone action at *α*v*β*3 may also regulate activity of Na, K-ATPase. A direct influence of change in [Na^+^]_*i*_ or [K^+^]_*i*_ on P-gp activity is not proposed, but inhibition by tetrac of the sodium pump will result in increased intracellular [Na^+^] and decreased [K^+^]. It is not known whether a specific change in intracellular [K^+^] or [Na^+^] affects P-gp, but inhibition by ouabain of Na, K-ATPase increases P-gp (MDR1) mRNA [45], suggesting that the monovalent cation microenvironment may directly or indirectly affect P-gp protein abundance. An indirect mechanism would be the effect of increased [Na^+^]_*i*_ to increase [Ca^2+^]_*i*_ by activation of the Na^+^-Ca^2+^ exchange in reverse mode [46], a factor that is relative to the discussion below of calmodulin.

Epidermal growth factor (EGF) can increase efflux activity of P-gp [25], apparently by phospholipase C-dependent phosphorylation of the pump. We have found that agonist thyroid hormone can enhance the biochemical activity of EGF [47, 48] and that tetrac blocks the capacity of thyroid hormone to potentiate EGF actions on signal transducing kinases. Thus, we expect tetrac to be capable of modifying the action of EGF on P-gp, favoring chemosensitivity. This possibility has not been experimentally tested. It is also important to note that transcription of the EGF receptor (*EGFR*) gene is inhibited by nanoparticulate tetrac [17, 30], so that the trophic effect of endogenous EGF on P-gp is unlikely to be manifested in the presence of nanotetrac.

VEGF can acutely decrease activity of P-gp, without a change in number of pumps/cell [26]. This is an interesting observation, indicating that anti-VEGF clinical strategies could increase chemoresistance of cancer cells. The action of VEGF on P-gp is decreased by nocodazole, an inhibitor of microtubule polymerization, suggesting that P-gp internalization or orientation might contribute to its efflux activity [49]. Tetrac and its nanoparticulate formulation are potent antagonists of VEGF actions by multiple pathways [16, 17]. Bevacizumab and aflibercept are VEGF-directed, clinical antiangiogenic agents that also relieve P-gp from VEGF-imposed inhibition. However, the inhibitory effect of VEGF is Src kinase-requiring [26] and tetrac is known to downregulate this kinase via the thyroid hormone/tetrac receptor on *α*v*β*3 [50]. Against this background, we may speculate that agonist thyroid hormone (T_4_ or T_3_) may support the action of VEGF on P-gp activity and chemoresistance, whereas tetrac and nanotetrac will oppose the effect.

OPN also increases cellular abundance of P-gp mRNA [24]. It does so via its interaction with the ectodomain of integrin *α*v*β*3. A clinical study has demonstrated that thyroid hormone increases OPN production [51]. Thus, it is possible that the thyroid hormone effect on P-gp may also have a contribution from increased availability of OPN for interaction with *α*v*β*3 that is thyroid hormone-directed. Hypoxia is another factor that serves to upregulate* OPN* gene expression [24] and thus may enhance chemoresistance.

Thyroid hormone (T_3_) increases expression of the hypoxia-inducible factor 1-*α* (*HIF-1α*) gene via *α*v*β*3 [50]; the* HIF-1α* gene product increases transcription of the P-gp gene [23, 52, 53]. The action of T_3_ on HIF-1*α* abundance is inhibited by tetrac [50]. Thus, thyroid hormone analogues may act on P-gp gene expression by more than one mechanism including the T_3_-nuclear thyroid hormone receptor (TR) pathway [8, 54] and also through control of HIF-1*α* production that begins nongenomically for T_3_ at *α*v*β*3.

A contribution of intracellular [Ca^2+^] to the function of P-gp is inferred by the effect of verapamil to decrease efflux pump activity. However, it is not clear that this action of verapamil relates to its prototypic calcium channel effects, since certain other channel blockers may not inhibit P-gp but can affect the multidrug resistance state of cells [55]. Thyroid hormone is a regulator of [Ca^2+^]_*i*_ via hormonal actions on plasma membrane Ca^2+^-ATPase (“calcium pump”) [39, 40]. This effect of thyroid hormone is dependent upon calmodulin. Verapamil has been shown by us to block the stimulatory effect of T_4_ on the calcium pump by interfering with the interaction of calmodulin with the ATPase [39]. Calmodulin is involved in control of P-gp activity through calmodulin-dependent kinase II activity [27, 28]. Thus, the conventional experimental use of verapamil to inhibit the P-gp axis may extend to calmodulin-relevant thyroid hormone actions that are linked to the efflux pump. It is not clear whether [Ca^2+^]_*i*_ has roles in modulation of P-gp activity or the actions of tetrac/nanotetrac on the efflux pump, beyond generation of calmodulin-Ca^2+^ complexes.

A mechanism does exist by which agonist thyroid hormone (T_4_ or T_3_) might decrease cell P-gp activity, as tetrac appears to do via *α*v*β*3. The hormone induces cellular reactive oxygen species (ROS) generation [56, 57] and this may reduce P-gp [58, 59]. One of the coauthors of the present paper (S Incerpi) has shown that integrin *α*v*β*3 is not involved in T_3_-directed generation of ROS in hepatocytes [57]. Control thyroid hormone-containing (FBS-supplemented) culture medium for tumor cells does not increase intracellular residence time of chemotherapeutic agents [9] that is clearly seen with exposure of cells to tetrac.

## 4. Discussion

The observation that tetrac/nanotetrac can chemosensitize tumor cells previously resistant to agents such as doxorubicin and etoposide [9] caused us to undertake the present review of molecular mechanisms that may be the basis for actions of tetrac/nanotetrac on P-gp. Tetrac/nanotetrac oppose the nongenomic actions of T_4_ and T_3_ at plasma membrane integrin *α*v*β*3 that regulate a variety of plasma membrane transport systems—such as the Na^+^/H^+^ antiporter, Na, K-ATPase, and Ca^2+^-ATPase [60]—that may be relevant to P-gp activity or to transcription of the* MDR1* (P-gp) gene. Further, integrin *α*v*β*3 interacts with OPN and with the VEGF/VEGFR axis, offering opportunities for thyroid hormone analogues to modulate the influence of OPN and VEGF on P-gp.  Table 1 summarizes a group of factors that modulate P-gp action and may be contributors to the increased intratumor intracellular residence time of chemotherapeutic agents in tetrac/nanotetrac-exposed tumor cells.

The most obvious molecular mechanism that contributes to the apparent effect(s) of tetrac/nanotetrac on P-gp is the action of these hormone analogues on intracellular pH. Tetrac acidifies cells by inhibiting the Na^+^/H^+^ exchanger and the P-gp efflux pump is arrested by an acid intracellular environment. Here, the importance of the generous expression of the agent's target—integrin *α*v*β*3 with the tetrac receptor—on cancer cells is critical, so that conventional and necessary activity of MDR1 in nonmalignant tissues is unimpaired in the presence of nanotetrac. Unmodified tetrac is unsatisfactory for cancer management because in the intact organism it is taken up by normal cells, as are T_4_ and T_3_. Within the normal cell, unmodified tetrac is a low-potency thyromimetic that can promote hypermetabolism.

The plasma membrane sodium pump and calcium pump are also regulated nongenomically by thyroid hormone. Inhibition of such nongenomic actions of thyroid hormone at *α*v*β*3 by nanotetrac would serve to increase [Na^+^]_*i*_ and [Ca^2+^]_*i*_. Such changes are not known to directly affect P-gp, although calmodulin-Ca^2+^ complexes are involved in calmodulin kinase-mediated effects that serve to increase P-gap activity, as mentioned above.

It is also apparent that P-gp and thyroid hormone analogues share mechanistic interests in a diverse set of protein molecules. As noted above, thyroid hormone increases transcription of the* OPN* gene and the OPN protein activates P-gp. Thus, in the clinical setting, host T_3_ (and T_4_ as a prohormone for T_3_) that acts via nuclear TR may support chemoresistance via P-gp. Nanotetrac is unlikely to affect P-gp via OPN because actions of nanotetrac are limited to *α*v*β*3 and do not directly involve TR [17].

In contrast, EGF stimulates P-gp activity [25] and we have shown that, acting at the cell surface, thyroid hormone can potentiate certain effects of EGF [47]. Acting nongenomically, tetrac can inhibit agonist thyroid hormone action on EGF. Thus, a component of the prolongation of intracellular residence time of certain chemotherapeutic agents in nanotetrac-exposed cancer cells may be due to blockade of the action of T_4_ at the EGF receptor.

Recent reviews of regulation of P-gp [1, 4, 5, 61] endorse the search for new approaches to the efflux pump that are suitable for application to clinical chemoresistance. New approaches are facilitated by characterization of previously unrecognized control mechanisms for P-gp. We point out here that integrin *α*v*β*3 offers access to multiple regulatory pathways for MDR1 that may be suitable for pharmacological exploration. We have emphasized in this review the potential usefulness of the cell surface receptor on *α*v*β*3 for thyroid hormone and tetrac/nanotetrac as a regulator of P-gp. However, the specific interactions of the integrin with extracellular matrix proteins, for example, OPN or growth factors, and existence on the integrin of other small molecule receptor sites offer new opportunities to modulate efflux pump activity.

Finally, it is interesting to note two additional interactions of thyroid hormone and P-gp. First, the export of the hormone from cells is a P-gp-mediated, verapamil-inhibitable process [62, 63] and thus to the extent that thyroid hormone may increase P-gp activity—or nanotetrac may inhibit such activity—intracellular hormone levels may be affected. For purposes of efflux, the hormone is a ligand of P-gp, but regulation by the hormone of P-gp activity is likely to originate at integrin *α*v*β*3 and involve intermediary kinases implicated in transporter control [41]. Second, the extensive intracellular trafficking of P-gp among compartments is actin-dependent [64]. The integrity of the actin cytoskeleton and maintenance of F-actin is in part T_4_-regulated [31, 32]. The nongenomic actions of thyroid hormone on intracellular protein trafficking are reviewed elsewhere [17, 65].

In summary, P-glycoprotein (MDR1; ABCB1) is a ubiquitous plasma membrane efflux pump capable of exporting specific pharmacologic agents. In tumor cells, P-gp substrates include chemotherapeutic agents such as doxorubicin, etoposide, and trichostatin A. Thyroid hormone is known to stimulate expression of the* MDR1* gene and activity of P-gp and thus may be seen to support chemoresistance. Tetrac is a thyroid hormone antagonist at the thyroid hormone-tetrac receptor on cell surface integrin *α*v*β*3 and exposure of tumor cells to tetrac desirably increases retention time of the cancer chemotherapeutic agents that are known substrates of P-gp. A variety of molecular mechanisms are reviewed here by which thyroid hormone/tetrac may influence P-gp activity. Of interest is that cisplatin is not a substrate of P-gp, yet we have shown elsewhere [9] that its intracellular residence time is also increased by tetrac, raising the possibility of multiple mechanisms by which tetrac affects tumor cell handling of anticancer drugs. For example, tetrac may increase the activity of the organic cation transporter (OCT) [66] that imports (rather than exports) cisplatin, perhaps by inducing intracellular acidosis via the Na^+^/H^+^ antiporter, as discussed above. Thus, “intracellular residence time” of drugs in response to tetrac may reflect decreased P-gp efflux or, possibly, increased cationic transporter influx. The latter mechanism has not yet been examined.

## Figures and Tables

**Table 1:** Selected intra- and extracellular factors that affect activity and/or abundance of P-glycoprotein (P-gp; MDR1).

Factor	P-gp activity	P-gp abundance	Reference
Intracellular pH (pHi)	↓	↓	[20]
Extracellular pH (pHe)	↑	NS	[21]
Hypoxia	↑	↑	[22]
Hypoxia-inducible factor 1-*α* (HIF-1*α*)	NC	↑	[23]
Thyroid hormone/analogues			
T_4_, T_3_	↑	↑	[6]
Tetrac/nanotetrac	↓	NC	[9]
Osteopontin (OPN)	↓	↓	[24]
Epidermal growth factor (EGF)	↑	NS	[25]
Vascular endothelial growth factor (VEGF)	↓	NC	[26]
Calcium channel blockers	↓	↓	[2]
Ouabain	NS	↑	[27]
Calmodulin antagonists E6, EBB	↓	NS	[28, 29]

T_4_: L-thyroxine.

T_3_: 3,5,3′-triiodo-L-thyronine.

NC: no change in parameter.

NS: parameter not investigated/recorded.

A variety of additional pharmacologic inhibitors of P-gp are reviewed in [3–5].
